# Social Mechanisms in Epidemiological Publications on Small-Area Health Inequalities—A Scoping Review

**DOI:** 10.3389/fpubh.2019.00393

**Published:** 2019-12-20

**Authors:** Kim Alexandra Zolitschka, Oliver Razum, Jürgen Breckenkamp, Odile Sauzet

**Affiliations:** ^1^Department of Epidemiology and International Public Health, School of Public Health, Bielefeld University, Bielefeld, Germany; ^2^Centre for Statistics, Bielefeld University, Bielefeld, Germany

**Keywords:** social mechanism, health inequalities, small-area effects, neighborhood effects, processes

## Abstract

**Background:** Small-area social mechanisms—social processes involving the social environment around the place of residence—may be playing a role in the production of health inequalities. Understanding how small-area health inequalities (social environment affects health and consequently contribute to inequalities between areas) are generated and the role of social mechanisms in this process may help defining interventions to reduce inequalities. In mediation and pathway analyses, social mechanisms need to be treated as processes or factors. We aimed to identify which types of social mechanisms explaining the process leading from small-area characteristics to health inequalities have been considered and investigated in epidemiological publications and to establish how they have been operationalized.

**Methods:** We performed a scoping review for social mechanisms in the context of small-area health inequalities in the database PubMed. Epidemiological publications identified were categorized according to the typology proposed by Galster (social networks, social contagion, collective socialization, social cohesion, competition, relative deprivation, and parental mediation). Furthermore, we assessed whether the mechanisms were operationalized at the micro or macro level and whether mechanisms were considered as processes or merely as exposure factors.

**Results:** We retrieved 1,019 studies, 15 thereof were included in our analysis. Eight forms of operationalization were found in the category social networks and another nine in the category social cohesion. Other categories were hardly represented. Furthermore, all studies were cross sectional and did not consider mechanisms as processes. Except for one, all studies treated mechanisms merely as factors whose respective association to health outcomes was tested.

**Conclusion:** In epidemiological publications, social mechanisms in studies on small-area effects on health inequalities are not operationalized as processes in which these mechanisms would play a role. Rather, the focus is on studying associations. To understand the production of health inequalities and the causal effect of social mechanisms on health, it is necessary to analyze mechanisms as processes. For this purpose, methods such as complex system modeling should be considered.

## Introduction

In many welfare states a high standard of living and a well-established social security system dominate. But since the 1990s, the difference in living conditions between the poorest and the better-off has increased (1). A link has been established between individual social economic status and health showing that a low socioeconomic status (measured e.g., by education, income, and occupational position) leads to an increased occurrence of risk factors, symptoms, diseases, and premature death (1, 2).

**Figure 1:**
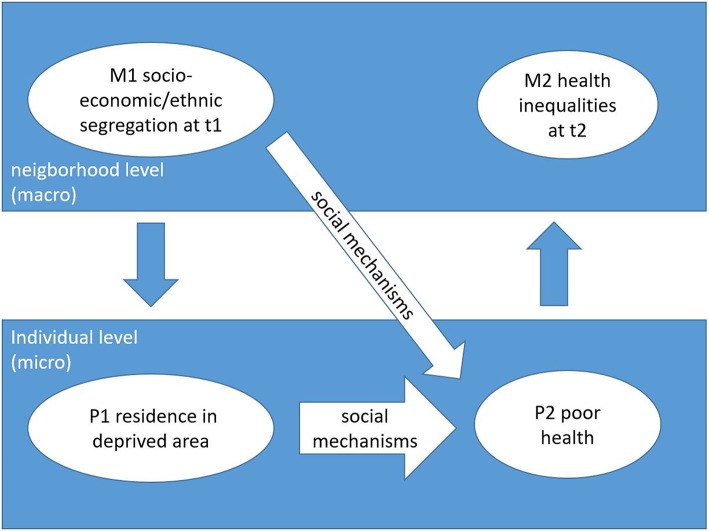
Schematic representation for the concept of different level mechanisms regarding health inequalities [adapted from Hedström and Swedberg (11)].

In addition to individual socio-economic factors, there is growing evidence that mechanisms at small-area level (also known as neighborhood effects) play a role in the production of health inequalities (3–5). Small-area is a general term used in the literature alongside neighborhood. Small-area means a geographical unit including the place of residence which can be of any size or cover different types of administrative units. Wilson's book “*The truly disadvantaged: The inner city, the underclass, and public policy”* (6) was one of the first publications (1987) regarding small-area health inequalities. The small-area context (structures in the social and physical environment where individuals live) influencing health can contribute to health inequalities between areas (6). While environmental mechanisms (e.g., air pollution) are well-studied in epidemiological literature, social mechanisms (e.g., changing health through healthy behavior encouraged by the behavior of neighbors) are not (7). For this reason our research focus on social mechanisms. We consider two types of concepts of social mechanisms relevant to the context of small-area health inequalities. Other mechanisms influencing health inequalities (e.g., environmental mechanisms) are not included in our examples. The first concept highlights the presence of micro and macro levels. The micro level corresponds to the individual level and the macro level to the small-area (or collective) level (8). This approach follows the principle of macro-micro levels of Hernes (9) and Smith and Conrey (10) in which the purposive actions of individuals produce a joint social action. For the production of health inequalities, socio-economic stratification by place of residence (small-area) (M1, at *t*1, see Figure 1) and health inequalities (M2, at *t*2, see Figure 1) are on the macro level. Social mechanisms operating at the micro level, leading from residence, say, in a deprived area (P1) over time *t*1–*t*2 to poor health (P2), and poor health leads to health inequalities relative to residence in a less derived area (M2).

Secondly, we consider a one-level concept, in which a mechanism is defined by its mediating role. The cause and the effect are both at micro level.

This concept reflects the statistical approach of pathway analyses: an assumption of causal pathways between factors can explain how independent factors may influences an outcome.

**Figure 2:**
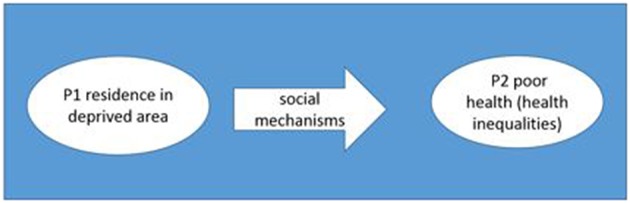
Schematic representation for the concept of one level mechanisms regarding health inequalities.

The phenomenon, health inequalities, develops through concatenations of causally linked factors (12). Residence in deprived areas (P1, see Figure 2) leads to poor health through social mechanisms (P2). The difference in health status (poor health and good health) can lead to health inequalities between areas.

A typology of mechanisms proposed in the sociological literature to explain small-area effects on a wide range of outcomes has been reviewed and categorized by Galster (13). These comprise four main types: social mechanisms, environmental mechanisms, geographical mechanisms, and institutional mechanisms; only the former are covered in our review. Social mechanisms which have been hypothesized to explain small-area effects include social networks, social contagion, collective socialization, social cohesion and control, competition, relative deprivation, and parental mediation (13). Social networks are interpersonal communications of information or resources by neighbors which can influence individual people. These networks consist of either strong ties, weak ties or both. Social contagion is the spread of ideas, attitudes, or behavior patterns in a group through imitation and conformity (8, 13). In a city, mostly all inhabitants have social contacts and peers. The health behavior from these contacts can influence one's own health behavior or vice versa (14, 15). The mechanisms collective socialization enables the adaption of behaviors or attitudes from peers or neighbors. This adaption is due to neighborhood role models or social pressure. A minimum threshold or critical mass has to be achieved for a successful adaption. The social cohesion within a neighborhood (degree of social disorder or the converse) can influence individual behavior. Competition is a mechanism where groups within the neighborhood compete for certain limited, local resources among themselves. Access to these resources may be determined by the success of the own group. Relative deprivation means that residents with socioeconomic success may be a source of amenities for their disadvantaged neighbors. Parental mediation mirrors the influence of the home environment on the children (parents' health, parents' behavior, stress, coping skills, material resources).

Understanding small-area health inequalities, in particular the role social mechanisms play in their production, may help developing interventions or policies aiming at reducing inequalities (16). Social mechanisms can either be treated as processes within a two-level framework or within a one level framework as factors within a pathway. Epidemiological research is often based on analyses of associations between risk factors and health outcomes instead of analyzing processes.

With this review we aim first to identify which type of social mechanisms have been investigated in epidemiological publications regarding the influence of small-area -via social mechanisms- on individual health and thereby producing health inequalities. Second, we document how social mechanisms at small-area level have been operationalized (process, factor, how they were measured) in quantitative epidemiological studies.

## Materials and Methods

A scoping review was carried out to answer the following review question: which social mechanisms are used in epidemiological publications when investigating health inequalities in a small-area context?

Included in the review were studies from 1987 until September 2019 following the publication of Wilson's book “*The truly disadvantaged: The inner city, the underclass, and public policy”* (6). The main inclusion criterion is the use of the term social mechanism. With epidemiological publications we mean studies which investigate the distribution, determinants, and etiology of health outcomes in a population.

Due to limited financial capacities, the search was limited to English and German language publications. Studies of small-area effects on health which do not test at least one mechanism were excluded.

The literature search was limited to the database PubMed to increase the likelihood of retrieving epidemiological studies. The search was performed in September 2019 with the following search string:

Health [Title] AND (inequalit*[Title/Abstract] OR differential*[Title/Abstract] OR inequit*[Title/Abstract] OR disparit*[Title/Abstract] OR heterogeneities [Title/Abstract]) AND (neighborhood OR small-area OR space OR spatial*) AND (social [All Fields] OR sociology [All Fields])

Two reviewers independently selected the publications and performed consistency checks with the inclusion criteria.

Basic information (authors, numbers, countries, exposures, outcomes, and results) were collected. Furthermore, we collected the form of operationalization of social mechanisms (e.g., the mechanism social network can be operationalized by the number of friends) and then we categorized them according to the typology of mechanisms proposed by Galster (social networks, social contagion, collective socialization, social cohesion, competition, relative deprivation, and parental mediation) (13).

## Results

**Figure 3:**
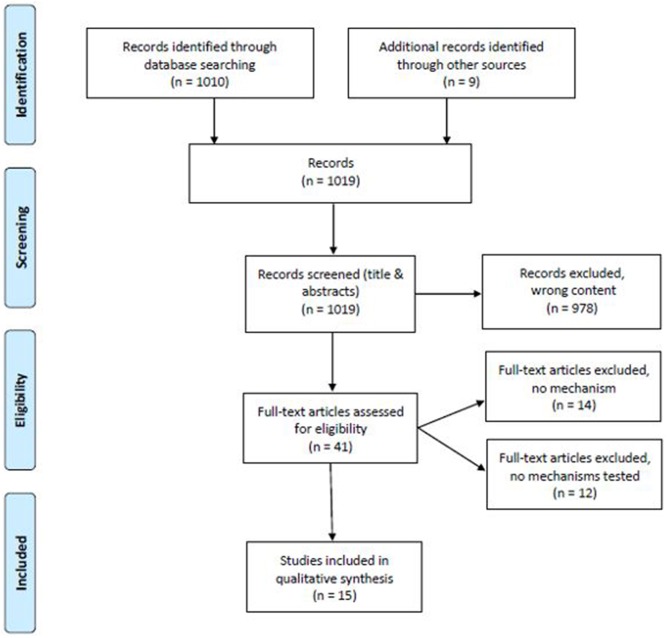
Flow diagram [modified according to Moher et al. (32)].

The search resulted in 1,010 publications. The publication characteristics are presented in Table 1. Nine additional studies were retrieved by reviewing the references of included studies/publications. Titles, abstracts and result sections were checked for consistency with the review question after which 978 studies could be excluded. Full texts of the remaining 41 studies were obtained and 14 studies were subsequently excluded because no mechanisms were mentioned. We also found 12 studies (not included in this review) which mentioned social mechanisms but did not directly quantitatively assess their effects on health inequalities. The remaining 15 studies were included in our analyses. Figure 3 summarizes the selection of literature in a flow diagram according to Moher et al. (32).

**Table 1:** Overview about study characteristics.

**References**	**Country**	**Study design**	**Outcome**	**Exposures**	**Results**
Ard et al. (17)	USA	Cross sectional	Self-rated health	Informal social participation, faith based social capital, political activism, general social trust, organizational social participation, formal group involvement, electoral politics	All measures are significantly related to self-rated health
Iwase et al. (18)	Japan	Cross sectional	Self-rated health	Parents and teachers association, sports club, alumni associations, political campaign clubs citizen's club, community associations (homogeneous or heterogeneous according to their social composition)	Heterogeneous exposures are inversely associated with poor self-rated health, women benefited more from heterogeneous and men more from homogeneous activities
Dahl and Malmberg –Heimonen (19)	Norway	Cross sectional	Self-rated health, longstanding illness	Emotional support, practical support, number of friends and acquaintances, neighborhood satisfaction, civic participation, own education and access to professional resources, generalized trust	Neighborhood satisfaction and generalized trust is positively associated with self-rated health
Pinxten and Lievens (20)	Belgium	Cross sectional	Self-rated physical health	Perception that respondents can live comfortably within their available income (economic capital), education, participation in cultural activities, participation in recreational activities, social support, neighborhood social cohesion	Low level of economic capital has a negative effect on mental health; more economic capital lead to better mental health; social support correlated positively with mental and physical health; neighborhood social cohesion is correlated positively to mental health; cultural participation has a positive effect on physical health
Gatrell et al. (21)	England	Cross sectional	Psychological morbidity (self-rated)	Material circumstances, loneliness, social cohesion, contact to neighbors	Presence of a person to trust leads to lower mental health and vice versa
De Clercq et al. (22)	Belgium	Cross sectional	Self-rated health	Individual social capital (participation in clubs, organizations), community social capital	Individual and community social capital are positively associated with perceived health
Chandola (23)	UK	Cross sectional	Self-rated health	Fear of crime	High fear of crime leads to poorer self-rated health
Bjornstrom (24)	USA	Cross sectional	Self-rated health	Relative position, trust	Trust is positively related to health
Fone et al. (25)	Wales	Longitudinal	Self-rated mental health	Social cohesion	Living in a medium or high social cohesion neighborhood is associated with a better mental health
Baum et al. (26)	Australia	Cross sectional	Self-rated physical health	Social network, social support, reciprocity, trust, neighborhood cohesion, neighborhood safety	High cohesion and high safety in nationhood lead to better self- rated health
Ziersch et al. (27)	Australia	Cross sectional	Self-rated mental and physical health	Neighborhood connection, neighborhood Trust, reciprocity, neighborhood Safety, local civic action	People with more positive perceptions of neighborhood safety have better physical and mental health. Additional people with strong neighborhood connections reported better mental health
Mitchell et al. (28)	UK	Cross sectional	Self-rated physical health	Peoples attitude to their community	People who do not feel part of their community are more likely to report a high number of symptoms
Boardman (29)	USA	Cross sectional	Self-rated physical health	Residential stability	Impact of stress on physical health is stronger among residents of unstable neighborhoods
Putrik et al. (30)	The Netherlands	Cross sectional	Self-rated health	Neighborhood safety, social cohesion	Residents who feel unsafe in their community were less likely to report good health and few depressive symptoms. People with low social cohesion were less likely to report good health
de Vries et al. (31)	The Netherlands	Cross-sectional	Self-rated health	Social cohesion	Social cohesion mediates between local greenspace availability and residents health

Basic information about these studies are provided in Table 1. Most studies (four) were from the UK (21, 23, 25, 28), followed by the USA (17, 24, 29). Two studies were from Australia (26, 27), two from Belgium (20, 22) and two from the Netherlands (30, 31). One study originated from Norway (19) and one from Japan (18).

All studies used self-reported measures of health as outcome. Seven studies had a measure of general health or aspects of health (17, 18, 22–24, 26, 31). One study was restricted to physical health (28) and two distinguished between mental and physical health (20, 27). Five studies focused on mental health only (19, 21, 25, 29, 30).

Fourteen studies aimed to show a statistical association between exposures (form of operationalizations of mechanisms) and outcome (self-reported health). One study (31) analyzed social cohesion as mediator between local greenspace availability and health. Furthermore, all studies were cross-sectional.

The forms of operationalizations of social mechanisms in the studies were classified according to the typology of Galster (13). We found 20 different forms of operationalizations of social mechanisms in the studies. No mechanism could be classified under the categories “collective socialization,” “competition,” “parental mediation,” and only one could be classified, respectively, under the categories “relative deprivation” and “social contagion” (see Table 2).

**Table 2:** Overview about social mechanisms, their forms of operationalization and measurement.

**Social mechanism [Galster (13)]**	**Form of operationalization**	**Measurement**
Social networks	Social support [(26), (20)^*^]	Five item scale that measured in which situations a person can rely on others (20), five-point scale based on respondents perception to ask others for help (five scenarios) (26)
	Emotional support (19)	Five item scale. People were asked questions according to emotional help from other people (19)
	Practical support (19)	Eight item scale with questions about practical help from others (19)
	Number of friends (19), neighborhood connections (27)^*^	Number of reported friends (19), four questions about neighborhood connections (27)
	Informal social participation [(17)^*^, (26), (18)^*^]	Index which was calculated by the mean of five questions (17), number of homogeneous groups people were active in (18), reported face-to-face contacts except housemates (26)
	Presence of a person to trust (21)^*^	Binary question (21)
	Norm of reciprocity [(26), (27)]	Question: “by helping others you help yourself in the long run” (26), number of favors given and received (27)
Social cohesion	Social cohesion [(25)^*^, (26)^*^, (20)^*^, (22)^*^, (30)^*^, (31)^*^]	Sub scale of Sampson et al. collective efficacy measure (20), five item scale (questions about neighborhood) (22), Buckner's' neighborhood Cohesion scale (25), five item question (26, 31) no information about measurement (30)
	Fear of crime (23)^*^, neighborhood safety [(26)^*^, (27)^*^, (30)^*^]	Question: “how safe do you feel walking alone in this area after dark?” (23), respondents rated their neighborhood on a scale from dangerous to safe (26), no information about measurement (30), two questions about neighborhood as a safe place to walk around at night and if people feel safe in their homes (27)
	General social trust [(26), (19)^*^]	Question: if “most people can be trusted or that you can't be too careful in dealing with people?” (19), trust of people in Australia, government, and big business (26)
	Neighborhood trust [(17)^*^, (24)^*^, (27)]	Index about generalized trust through different entities in the neighborhood (17, 27), question: how much “people in the neighborhood can be trusted” (24)
	Group involvement [(17)^*^, (26), (27), (20), (22)^*^, (19), (18)]	Question: participation in group activities e.g., Clubs or organizations (17–19, 22, 26, 27) question: participation in recreational activities (20)
	Neighborhood satisfaction [(19)^*^, (30)^*^]	Question: how satisfied are the respondents with their neighborhood (19)
	Residential stability (29)^*^	Two questions from respondents census tracts about residential stability (29)
	Attitude to community (28)^*^	Question: “do you feel part of the community”(28)
Social contagion	Cultural participation (20)	Question: participation in cultural activities (20)
Relative deprivation	Relative position (24)^*^	(ln(family income)-ln(MHI1))/ln (MHI1) (24)
Competition	–	–
Collective socialization	–	–
Parental mediation	–	–

* *Significant association between exposure and health outcome*. *MHI1 = tract level median household income*.

Eight forms of operationalizations belonged to the category “social networks”: social support, emotional support, practical support, number of friends/neighborhood connections, informal social participation, presence of a person to trust, norm of reciprocity, and social networks (see Table 2). A form of operationalization for the mechanism social support was found in two studies with a significant association in one study (20). Emotional and practical support were tested in one study, but the associations were not significant. We found “number of friends” in two studies and in one study the association was significant (27). Informal social participation was tested in three studies with significant findings in two studies (17, 18). “Presence of a person to trust” was reported in one study where the association was significant (21). Norm of reciprocity was found in two studies with no significance.

Eight forms of operationalization could be assigned to the category “social cohesion”: social cohesion index, fear of crime/neighborhood safety, general social trust, neighborhood trust, group involvement, neighborhood satisfaction, residential stability, attitude to community. Social cohesion was found in five studies and in all of them the association was significant (20, 22, 25, 26, 30, 31). Fear of crime was tested in two studies and perceived neighborhood safety in two studies as well. All forms of operationalizations showed a significant association with health outcomes (23, 26, 30) except for one study (21). General social trust and neighborhood trust were tested in five studies, three associations thereof were significant (17, 19, 24). We identified group involvement in seven studies. The association was significant in two studies (17, 22). Both residential stability and attitude to community were found in just one study, respectively. In each study the association was significant (24, 28). The effects of neighborhood satisfaction was tested in two studies. In both studies the association was significant (19, 30).

For the categories “social contagion” and “relative deprivation,” respectively, one form of operationalization (cultural participation and relative position) were identified, but only relative position showed a significant association with health (see Table 2). Only one study investigated a mechanism operationalized both on macro and micro level (30). The other studies were limited to the individual level. One study used pathway analysis (27) and another study mediation analyses (30) to assess the role of social mechanisms in a casual pathway thus using a one-level concept of social mechanisms (Figure 2). The other quantitative studies considered mechanism only as an association between a factor and a health outcome via regression models. Neither a two-level nor a one-level-concept of mechanism were thus considered. The forms of operationalization are shown in Table 2. Social networks and social cohesion have been mostly measured by direct questions or questionnaires from which indices were calculated. Two different validated scales have been used for the measurement of social cohesion (20, 25).

## Discussion

In this review we aimed to identify which types of social mechanisms explaining the process leading from small-area characteristics to health inequalities have been considered and investigated in epidemiological publications. Furthermore, we aimed to establish how these mechanisms have been operationalized in quantitative studies. We chose to classify them according to the typology proposed by Galster (13).

We found 15 epidemiological publications in which an analysis of the effect on health of at least one social mechanism is presented. Most studies (10) we found in the epidemiological literature used social capital as theoretical concept from which social mechanisms were derived and applied to health outcomes. Theories of social capital cover social mechanisms but do not directly address them. Social capital is a social theory which focuses on the normative cohesion of groups and on the mutual interaction between these and individuals (19). The social capital theories (or aspects of it) used were either from Putnam or Bourdieu, with a clear continental divide: European studies related to Bourdieu and North American ones to Putnam (33, 34). Bourdieu defined social capital as the individual ability to access potential social resources through biography and social network (33). Putnam's approach involves a more collective view on social capital. He defined social capital as “features of social organization, such as trust, norms, and networks that can improve the efficiency of society by facilitating coordinated actions” (34). The other studies investigated associations of interest without an explicit theoretical background.

In our review the mechanisms in Galster's categories social networks and social cohesion have been the most studied so far. Social networks were found in eight different forms of operationalization. Social cohesion was also found in nine different forms of operationalization.

A negative impact of the disparities in methods of measurement of similar mechanisms is that it limits the possibilities of comparison across different studies. The complexity of operationalizations of social mechanisms varied. Often the mechanisms were operationalized with simple questions and their validity remains unclear. Two studies used validated scales to operationalize their mechanisms (25, 26). The wider use of standard measurement of social mechanisms could help obtaining more comparable evidence.

Other social mechanisms (collective socialization, social contagion, competition, relative deprivation, parental mediation) present difficulties in terms of operationalization as mechanisms making data collection more complex. Consequently, these mechanisms are rarely operationalized. A reason for this apparent difference in interest may be that social mechanisms come in different degrees of abstraction. Social networks and social cohesion can be measured as factors (e.g., the mechanism social network can be measured as factor: number of friends) whereas most of the other mechanisms (collective socialization, social contagion, and parental mediation) are processes and cannot be analyzed as factors.

Social networks and social cohesion have been mostly measured by direct questions or questionnaires from which indices were calculated. This makes their use in epidemiological studies relatively easy. However, these forms of measurements provide data at the micro level only. Operationalizing social mechanisms at macro level as well as at micro level would enable the investigation of a two-level mechanism framework in which social mechanisms operate at both levels (11). Only one study considered mechanisms (social cohesion and general feeling of safety) on both, the micro and macro level (30). The lack of measurement at macro level may be showing the difficulty in the analyses of small-area inequalities of assessing what is the relevant small-area scale at which small-area level factors or mechanism should be (34, 35). For small-area health inequalities pragmatic approaches based on administrative areas are unlikely to be of relevance to social mechanisms. A more relevant small-area scale for social mechanisms may be an entity which revolves around an individual and include their daily social contacts.

The social mechanisms found in epidemiological publications seem so far to be merely treated as risks factors in association analyses. However, mechanisms need to be understood as processes and can only be evaluated in a dynamic setting in which health inequalities come to existence (9, 36–38). Treating social mechanisms only as factors could be too restrictive to fully understand the production of health inequalities especially the intertwining of macro and micro level. A complex system modeling approach as advocated by the Network on Complexity, Inequality and Health (39) would enable a better understanding of the production of health inequalities on both macro and micro level. They identified features which are necessary for the analysis of health inequalities thus should be adapted for small-area health inequalities.

Complex system modeling should include (39): (a) capturing of outcomes produced by many interacting variables; (b) analyzing a population while taking heterogeneous individuals into account; (c) allow dynamics from individuals interacting in different social subgroups and social networks; (d) capture dynamic interacting, casual influences including positive and negative feedbacks; and (e) consider stochasticity.

Kaplan et al. proposed agent-based and microsimulation models or system dynamic models to analyze the production of health inequalities (39).

One study used pathway analyses (27) and one study analyzed social cohesion as mediator (31) and thus tested the mediating role of the form of operationalization of social mechanisms on health. This studies analyzed small-area associated measures only on micro level according the concept of one-level mechanisms (Figure 2). All identified studies were cross-sectional and therefore no assessment of causality could be made. The analyses of processes require longitudinal studies and possibly a life course approach to identify the social mechanisms involved in critical periods (40). Effects of risk factors in the life course could be analyzed using complex system modeling. Social mechanisms are understood as processes and in the context of interest, processes leading to health inequalities. These mechanisms (e.g., social contagion or social cohesion) are part of pathways which may lead to small-area health inequalities over time. Most studies did not discuss how the mechanisms lead to health inequalities. Rather they focus on an association between the operationalized factor and a health outcome.

## Limitations

We used the typology of social mechanisms proposed by Galster (13). It may not be the most relevant here as it has not been specifically developed for the study of health inequalities. The choice of typology, however, is unlikely to influence the results because our main result relates to the study design rather than the typology.

We focused our review around social mechanism and restricted our search on health inequalities. This restriction may not cover the whole spectrum of studies on social mechanisms in epidemiological publications. But health inequalities are a central theme in social epidemiology and our review contains publications which attempt to evaluate social processes as part of a causal pathway between small area and health inequalities.

## Conclusion

In epidemiological publications, social mechanisms in studies on small-area effects on health inequalities are not operationalized as processes in which these mechanisms would play a role. The focus remains so far on studying associations between individual perception of social factors and health outcomes. To operationalize at macro level, the relevant small-area scale must be known but this remains work in progress.

To understand the production of health inequalities and the causal effect of social mechanisms on health it is necessary to analyze mechanisms as processes. For this purpose methods such as complex system modeling should be considered.

## Author Contributions

OS and KZ conceived the study and conducted the literature search. KZ wrote the first draft of the paper. OS, JB, and OR revised the draft. All authors contributed to the interpretation of the study results and approved the final manuscript.

### Conflict of Interest

The authors declare that the research was conducted in the absence of any commercial or financial relationships that could be construed as a potential conflict of interest.
